# Tatajuba: exploring the distribution of homopolymer tracts

**DOI:** 10.1093/nargab/lqac003

**Published:** 2022-02-02

**Authors:** Leonardo de Oliveira Martins, Samuel Bloomfield, Emily Stoakes, Andrew J Grant, Andrew J Page, Alison E Mather

**Affiliations:** Quadram Institute Bioscience, Norwich Research Park, Norwich NR4 7UQ, UK; Quadram Institute Bioscience, Norwich Research Park, Norwich NR4 7UQ, UK; Department of Veterinary Medicine, University of Cambridge, Madingley Road, Cambridge CB3 0ES, UK; Department of Veterinary Medicine, University of Cambridge, Madingley Road, Cambridge CB3 0ES, UK; Quadram Institute Bioscience, Norwich Research Park, Norwich NR4 7UQ, UK; Quadram Institute Bioscience, Norwich Research Park, Norwich NR4 7UQ, UK; University of East Anglia, Norwich Research Park, Norwich NR4 7TJ, UK

## Abstract

Length variation of homopolymeric tracts, which induces phase variation, is known to regulate gene expression leading to phenotypic variation in a wide range of bacterial species. There is no specialized bioinformatics software which can, at scale, exhaustively explore and describe these features from sequencing data. Identifying these is non-trivial as sequencing and bioinformatics methods are prone to introducing artefacts when presented with homopolymeric tracts due to the decreased base diversity. We present tatajuba, which can automatically identify potential homopolymeric tracts and help predict their putative phenotypic impact, allowing for rapid investigation. We use it to detect all tracts in two separate datasets, one of *Campylobacter jejuni* and one of three *Bordetella* species, and to highlight those tracts that are polymorphic across samples. With this we confirm homopolymer tract variation with phenotypic impact found in previous studies and additionally find many more with potential variability. The software is written in C and is available under the open source licence GNU GPLv3.

## Graphical Abstract

**Graphical Abstract:**
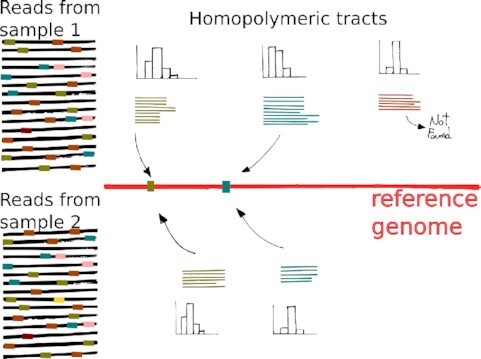
For each sample, represented by its reads, tatajuba finds homopolymeric tracts identifiable by their flanking regions, and maps them into a reference genome. It then compares the tracts mapped to the same locations in the reference.

## INTRODUCTION

The presence of repetitive DNA bases across bacterial genomes is ubiquitous and is associated with important phenotypic changes, especially in organisms with skewed GC content (1,2). These repetitive regions are known as homopolymeric. Since frameshifts are facilitated by such homopolymeric tracts (HT), they can lead to phase variation; the resultant change can lead to truncation of coding sequences with consequent changes in gene expression and therefore phenotype. Or if the HT occurs in non-coding control regions, it can affect the expression of genes. Identifying and monitoring all HTs in a sample can be challenging due to the large numbers and difficulty in identifying such tracts from sequence data. Since these frameshift events can be common in a population and regulate, or affect, the expression of genes with important phenotypic traits, effort should be directed to identify variation in tract lengths.

To date, evolutionary analyses have focused on single nucleotide polymorphisms (SNPs) and insertions and deletions (indels). In contrast to SNPs, HTs are harder to sequence, depend on the GC-content and may lead to biased coverage by current sequencing technologies (3–6). Furthermore, they are problematic to align across samples, since HTs are represented as indels (insertion-deletions) in phylogenetics. With the exception of a few specialist models (7–10), indels are treated as missing data in evolutionary inferences (11–13). They are furthermore challenging to catalogue, unless there is a specific region of interest which can then be curated manually for the presence of HTs. Our proposed algorithm, implemented in the software tatajuba, can be applied to any genome with an available annotated reference sequence, and can extract all HTs within the genome while allowing for tract length polymorphism even within a sample. To account for sequencing errors, tatajuba conservatively only calls HTs in areas with high read coverage, supported by both strands, and with sufficiently long flanking DNA on each read. The flanking regions must allow for the HT to be uniquely mapped to the reference genome, with length defined by the user, currently limited to less than 32 bp on each side. We consider read coverage in both forward and reverse strands by using a canonical representation of the tracts. The software can optionally be configured at runtime to be less conservative, neglecting the strand bias around HTs, with the risk of an elevated false positive rate of HTs identified or losing the ability to map against the reference in a few cases.

Our objective is to fully describe the distribution of tract lengths for all HTs in a sample, and to compare differences in HT length across a given set of samples. Differences in the variability of HTs across samples can give us information about evolutionary processes (diversity) and phenotype, and can be explicitly modelled. Changes in the tract length can suggest a phenotypic impact, and by providing BED and VCF files specific to the tracts, tatajuba facilitates exploring the functional impact of the HT variants. Using the whole distribution across reads, as opposed to assuming a single consensus sequence per sample, allows us to account for minor variants and intra-sample diversity, essential for observing small-scale evolutionary trends and phase variation in clonal populations (14).

We demonstrate the software capabilities on two data sets where the importance of phase variation has been previously described, *Campylobacter* (2) and *Bordetella* (15), but this tool can be applied to any microbial species. The name tatajuba (spelled tatajubá in Portuguese) comes from the South American hardwood tree *Bagassa guianensis*, meaning ‘yellow fire’ or ‘fire wood’ in Tupi-Guarani.

## MATERIALS AND METHODS

The software focuses on describing the tract length distributions across samples, by mapping them to a reference annotated genome and finding those with variability across samples. Given a FASTQ file as input, an HT is found from sequencing reads of nucleotides, and is defined as the same base repeated (e.g. 3 times or more), and flanked by a pair of k-mers, called ‘contexts’. Each context is thus a short DNA segment, between 10 and 32 bp typically, which flanks an HT and contains a rich set of nucleotide sequences. Using these flanking regions to anchor the HT avoids bioinformatic artefacts usually inherent around HTs. We will refer to an HT together with its pair of contexts simply as a tract, and we will consider only those tracts that can be mapped to the reference genome – otherwise they are discarded, but can be reported to the user since a large fraction of those can be indicative of an inappropriate choice of reference sequence or sequence contamination.

Furthermore, in the presence of paralogs or, specifically, when the exact same tract is mapped to more than one region in the reference genome, the method chooses the first one in genomic coordinates. To avoid inclusion of sequencing errors, we only consider an exact DNA sequence (i.e. read segment with identical contexts and homopolymer) observed in more than a number of reads fixed by the user (default is 5). Afterwards we can merge these identical DNA segments into a tract if their contexts are similar enough (based on their edit distance) and map to the same location in the reference genome, and their homopolymers are composed of the same base (see Table 1). Specifically, we use the C functions from BWA-aln for single ended reads (16) to map each tract against the reference. Some tracts will not be mapped due to inappropriate reference or contamination, and these are excluded from further analysis. In both cases the tract is not represented in the reference genome of choice (but may map to a different reference). The tracts might also fail to map due to poor aligner performance on low complexity regions, but large flanking regions should minimize this risk.

**Table 1.** The tract can be variable

DNA segment	Difference from canonical tract
**ATTCATCTAT**CCCCC**ATATCATTGA**	Canonical tract
**ATTCATCTAT**CCC**ATATCATTGA**	Tract length polymorphism
**ATTCATCTAT**CCCCC**ATATC*G*TTGA**	Substitution on right context
**TTCATCTAT*G***CCCCC**ATATCATTGA**	Insertion in left context
**TCAATGATAT**GGGGG**ATAGATGAAT**	DNA segment is reverse complement of canonical

All the following read segments come from the same tract, represented at the top as the ‘canonical’ or exemplary tract (notice that the contexts have fixed size of 10 bases in this example).

The tracts are therefore comparable across samples, where we can now create a list of tracts present in at least one sample (with an equivalent region also in the reference). In addition to reporting all identified tracts, we also highlight those that present variability across samples or in relation to the reference genome. This will generate a smaller set of tracts with potentially important biological implications. The measure of dispersion used here to find variable tracts is the absolute range (MAX-MIN). Other measures could be used, for instance the *relative difference of ranges* (similar to the coefficient of range), but it is being used here solely to exclude the tracts that do not change at all between samples. Tracts which are missing from a sample (but found in others) are also considered variable.

Besides the read files for all samples to be analysed, tatajuba requires the reference genome both in fasta format and its GFF3 file, such that it can access the annotations harbouring each tract and identify if the tract falls within a coding sequence or not. It works with prokka's GFF3 output (17), which means that the GFF3 file (i) can contain the fasta sequences, and (ii) can have more than one contig/chromosome/genome. Therefore, a multiple sequence FASTA file can also be provided in addition to the GFF3 file, which renders tatajuba compatible with multiple reference genomes.

For the phylogenetic comparison, we used snippy (https://github.com/tseemann/snippy) for generating the core genomes, assuming the same (single) reference genomes as for tatajuba. Using snp-dists (https://github.com/tseemann/snp-dists) to generate a distance matrix between the genomes considering only SNPs, we calculated the UPGMA trees with the hclust() function for R (18,19). For estimating the variant effects, snpEff was used after normalization and merging with bcftools of the VCF files output by tatajuba (20,21).

### Software implementation

The Levenshtein distance is used to decide if contexts from read segments represent the same tract. There are a few cases where the program detects two tracts that map to the exact same location in the reference genome. These cases may reflect a substitution within the homopolymeric region, which renders the flanking regions too dissimilar to be merged by the program. It can also happen when the flanking region includes an HT itself. Two examples are given in Table 2, where the same sample can present both versions of the tract.

**Table 2.** Examples where tatajuba finds more than one tract mapping to the same location in the reference genome

Tracts mapping to position 1269169 (cds-WP_002877328.1)
GGTGTTTTTAAGATGATAAGCATGC**TTT**GGGTCAGCAAGTGAAGAATTGACAC ← reference
GGTGTTTTTAAGATGATAAGCATGC**TTT**GGGTCAGCAAGTGAAGAATTGACAC ← tract tid_112808
TGGTGTTTTAAGATGATAAGCATGC**TTT**GG*A*TCAGCAAG*C*GAAGAATTGACAC ← tract tid_112807
Tracts mapping to position 127480 (unannotated)
TTTCTTACTAAAATATCCTTTGTAG**TTTT**ATCATTTCTTAAAACAAATTTCATT ← reference
TTTCTTACTAAAATATCCTTTGTAG**TTTT**ATCATTTCTTAAAACAAATTTCATT ← tract tid_113353
CTTACTAAAATATCCTTTGTAG*TTG***TTTT**ATCATTTCTTAAAACAAATTTCATT ← tract tid_113354

The polymeric tract is represented in bold, and differences in context (flanking regions) are highlighted in italics. The top panel shows an example where substitutions on the flanking regions and a (HT-related) deletion are responsible for the classification, while the bottom panel shows potentially successive insertions, with the ‘G’ disrupting the otherwise increased poly-T.

The maximum distance allowed can be controlled by the user, with the default being one, but the example above highlights how this information might be useful. By using the distribution of lengths in contrast to their consensus value, we can observe subtle changes in the populations, as for instance a sample where for a particular tract most reads have a length 4 homopolymer, but a few have length 5.

### Samples

It has been shown that the HT variation can be used to identify particular phenotypic traits, such as cell shape in *Campylobacter jejuni* (2). We thus analysed 100 *Campylobacter* samples used in (2): 68 of which had a phase variation described in one of the two genes of interest (*pgp1* and *pgp2*), and 32 ‘wild type’ samples, i.e., without a phase variation described in the original paper (list of samples and accession numbers available as Supplementary Tables and at https://github.com/quadram-institute-bioscience/tatajuba). We used *C. jejuni* M1 (ASM14870v1) as the reference genome.

Another study described a set of HTs with potential biological relevance and variability across three *Bordetella* species (15). In this study there were no available data to evaluate intra-species variation, but recently many data sets have been deposited in public repositories. We therefore were able to analyse 108 *Bordetella* samples downloaded from ENA (91 *Bordetella pertussis*, 7 *Bordetella parapertussis* and 10 *Bordetella bronchiseptica*), using *B. pertussis* Tohama I (ASM19571v1) as the reference genome. The *B. pertussis* samples come from bioprojects PRJNA348407, PRJNA356412, PRJEB42353 and PRJEB38438 (22–24), while the *B. parapertussis* and *B. bronchiseptica* come from bioproject PRJNA287884 (25). In order to analyse the 108 *Bordetella* samples, besides the single reference genome we also used a panel of reference genomes composed of a *B. pertussis* Tohama I (ASM19571v1), a *B. parapertussis* 12822 (ASM19569v1) and a *B. bronchiseptica* RB50 (ASM19567v1) for which we have the assembled and annotated genomes.

## RESULTS

**Figure 1.**
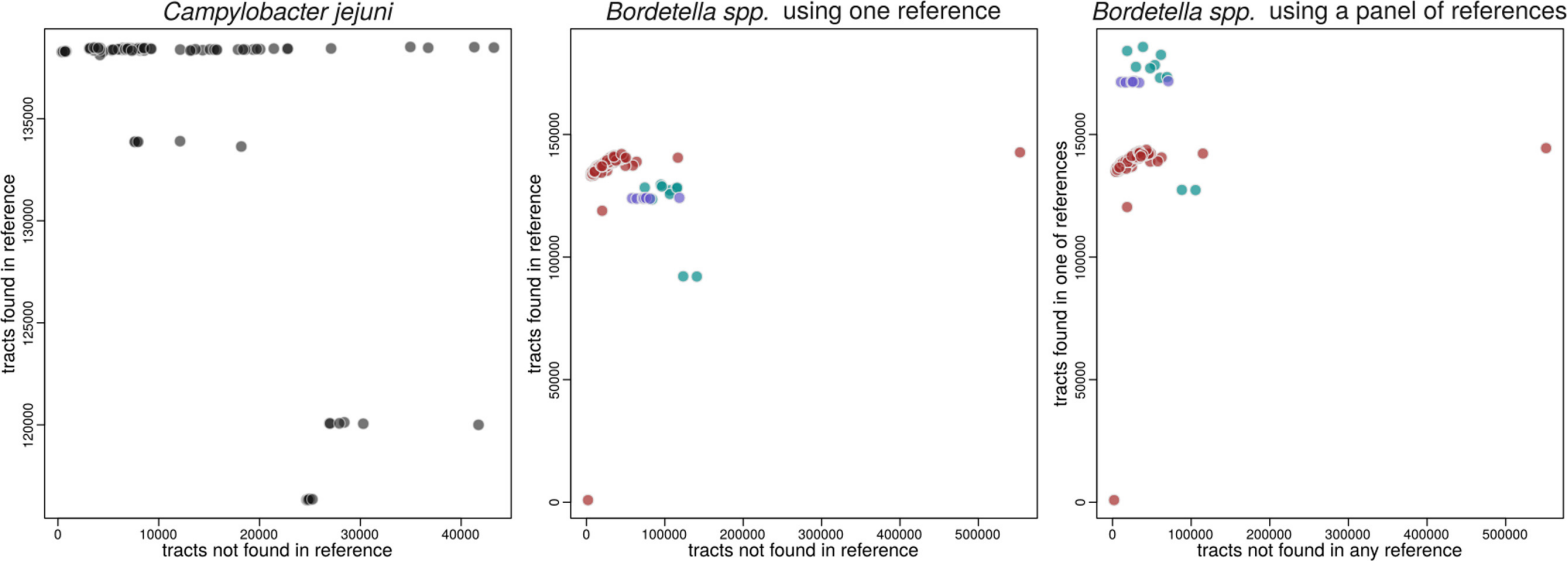
Number of tracts that could be mapped or not to the reference genomes per sample, for the *Campylobacter* (left) and *Bordetella* (middle) datasets using one reference genome, as well as for the same Bordetella dataset but using a panel of three reference genomes (right). Each point represents a sample, where some HTs can be mapped back to the reference genome (y axis) and some HTs cannot be mapped (x axis). The total number of HTs found by tatajuba are the sum of the x and y axes for each sample. For the *Bordetella* datasets (middle and right panels), the colours represent samples from different species (red for *B. pertussis*, green for *B. bronchiseptica*, and purple for *B. parapertussis*).

From each sample, we selected all tracts with homopolymeric lengths of 3 bp or more, which were present in at least 8 reads. We assumed a context of 28 bp on each side, and merged those with a Levenshtein distance smaller than 2, correcting for strand bias by removing HTs not observed in both directions. For these parameters, we found a total of 179 691 tracts for *Bordetella* and 144 119 tracts for the *Campylobacter* data set, which could be mapped to their single reference genomes. If we use a panel of reference genomes, then the number of tracts found for the *Bordetella* data set jumps to 496 293. The numbers for each individual sample are shown in Figure 1, where we can see that (i) both single reference data sets have comparable numbers of mapped HTs: around 140k tracts for most samples for both data sets, although the variation is higher for *Bordetella*; (ii) when a panel of references is used, several samples increase their number of mapped tracts, showing the benefit of adding references similar to the samples; (iii) however, even when using the wrong species (Figure 1, middle panel) the *B. parapertussis* and *B. bronchiseptica* did have more than half their HTs mapped to the *B. pertussis* reference, indicating some robustness to the reference choice; (iv) the unmapped tracts are a combination of the underlying evolutionary processes (e.g. novel mutations) and artefacts like technical contamination or poor choice of the reference genome, as we saw above from the inclusion of more references. Another artefact is confirmed by running the software without strand bias correction, where the number of unmapped tracts increases without an improvement on the number of mapped reads (result not shown). The exception is sample ERR4176311, a *B. pertussis* Tohama I delta-BP3063 mutant from (24), where almost all tracts are seen in only one strand: the 139 348 tracts mapped to the reference decrease to only 875 once we remove tracts not seen on both strand directions. This sample was removed from subsequent analyses. When the genome sizes are taken into account (1.6Mb for *Campylobacter* against 4Mb of *Bordetella*) then the frequency of HTs in *Campylobacter* is almost twice as high as in *Bordetella*.

**Figure 2.**
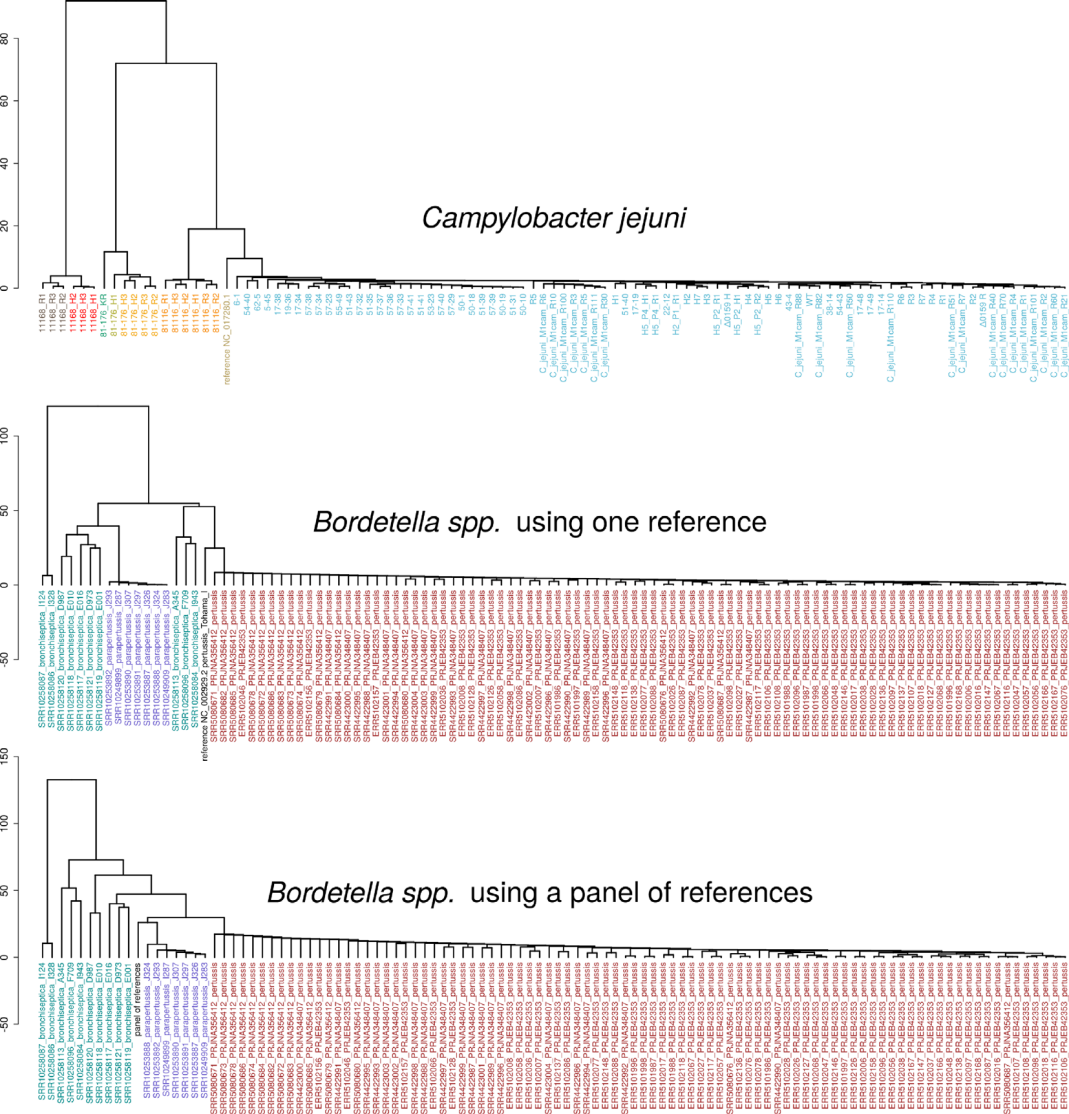
UPGMA dendrograms based on tract length profile similarity, using the average tract length as a feature. At the top, we have the dendrogram for the *Campylobacter* samples, with samples coloured according to the clustering. The middle and bottom dendrograms show the *Bordetella* samples, if we use one reference genome (*B. pertussis*) or a panel composed of three reference genomes, respectively. The colours for *Bordetella* represent the species as in Figure 1, with the tract length profile for reference genome or for the panel of three references in black.

Tatajuba analysed the 100 *Campylobacter* samples in 12 min and the 108 *Bordetella* in 15 min (single or multi-reference), using a computer with 48 cores and using less than 30GB. Currently we exclude tracts that cannot be mapped to the reference. In the *Campylobacter* dataset, we found 41 108 variable tracts (with variable length distributions or missing from one of the samples), of which 36 605 were annotated, that is, belonged to a gene or RNA. From the *Bordetella* data set, after removing sample ERR4176311 and using a single reference genome, we found 123 686 variable tracts, 106 206 of which were in annotated regions. By using the average length of an HT as a feature, we can cluster all samples based on how similar their sets of tracts are (in terms of their average length profile). The results are shown in Figure 2, where we see that such clustering is in agreement with the isolate names for Campylobacter, which indicate their strains M1, NCTC11168, 81116 or 81–176 (2). And in particular when using a panel of references, the Bordetella samples are monophyletic. We will discuss more about the phylogenetic agreement in the next section.

**Figure 3.**
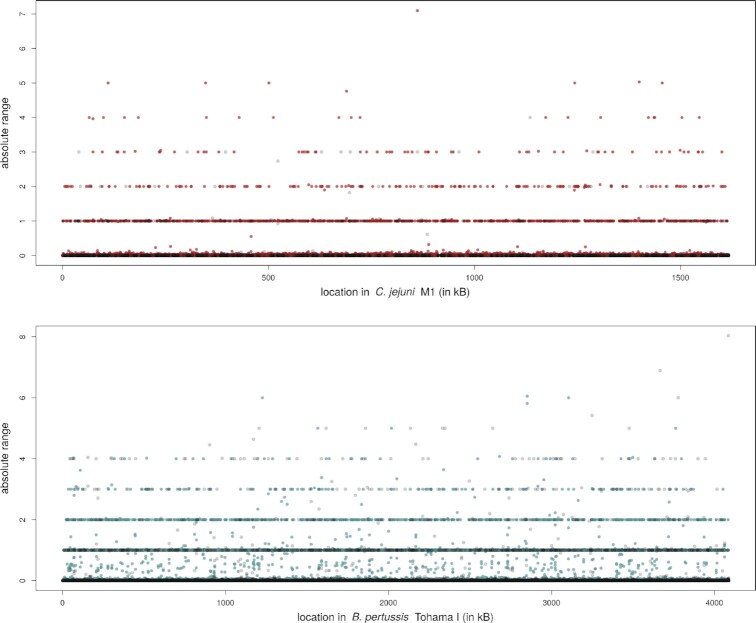
Tract length ranges (maximum minus minimum values across samples) across the reference genome of the two example data sets. Blue or red dots represent tracts in an annotated region while grey dots are not annotated. The tract length is estimated through the average length over reads, and only variable tracts are shown (i.e. those with range higher than zero).

We furthermore measured the variability of each tract across samples, and visualized them along the genome (Figure 3). In this figure, the variability is represented as the maximum difference between tract lengths across samples, where we can see it is not unusual to observe tracts where samples have a length difference higher than 4, for instance. This dispersion measure is currently used only to exclude HTs with no variation at all across samples, but as we see it gives an overview of highly variable tracts. Other measures can be implemented, which are more robust to outliers as for instance the interquartile range, or are based on the length distribution within a sample instead of a point estimate as cross-bin distances (26). Furthermore, length changes in multiples of three (a codon) might have a lower impact than those potentially changing the reading frame.

**Figure 4.**
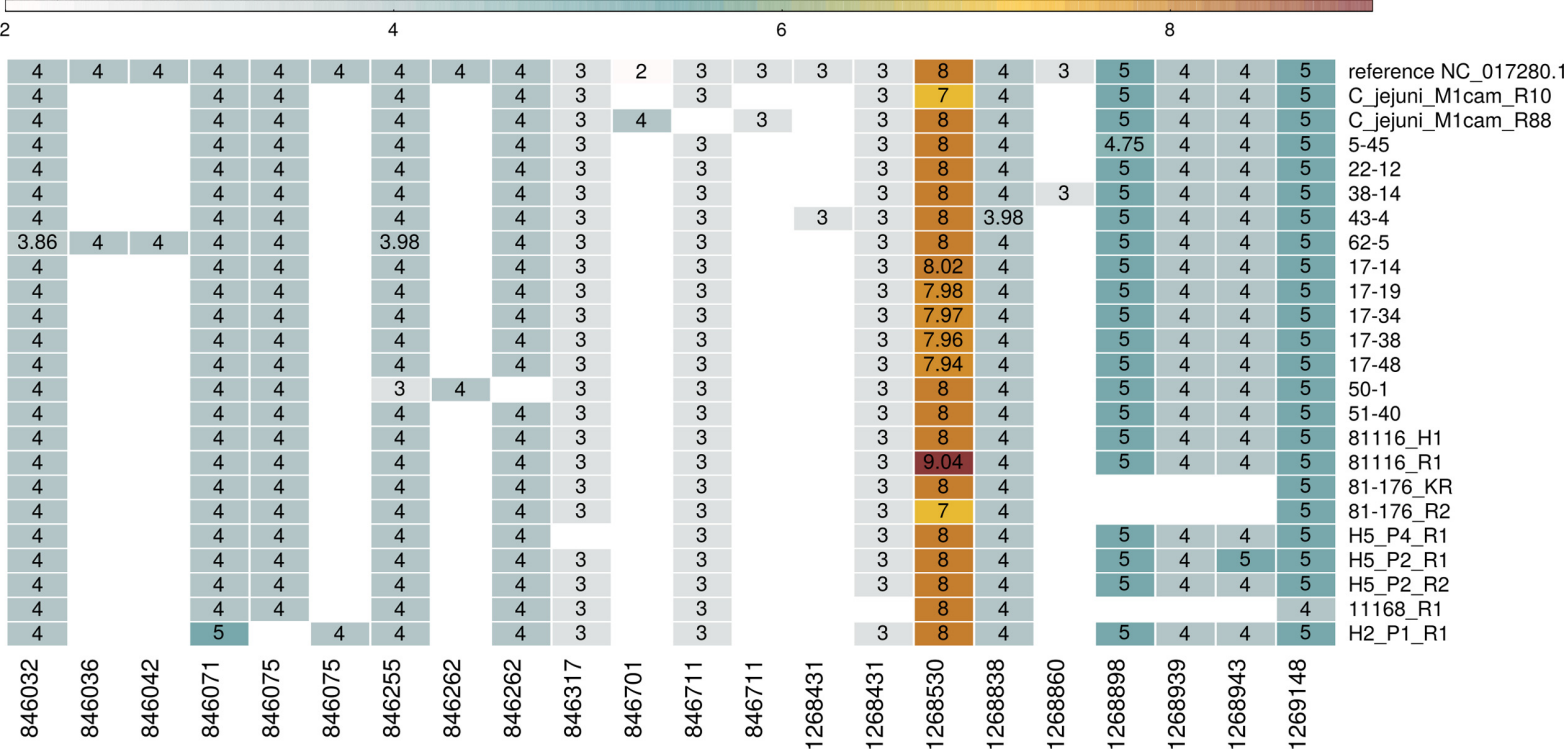
Average tract length for selected *Campylobacter* samples, over genes *pgp1* (from 1268323 to 1269717) and *pgp2* (846020 to 846997) for tracts described in (2). Rows correspond to samples, and columns are the genomic location of the HTs—only variable tracts are shown, i.e. if a tract has same length over all samples, then its column is excluded. The same location appears more than once for cases where tatajuba decides that the contexts are too distinct even if mapping to the same location in the reference genome. Tract lengths smaller than three were not considered by tatajuba and therefore are absent (empty cells).

In (2), 18 modifications in genes *pgp1* and *pgp2* were described which were associated with rod-shaped *C. jejuni* (Table 1 of that paper). Of these, 12 involve a change in the length of the homopolymer tract. In Figure 4 we observe that most mutations (10 out of 12) previously described leading to tract length modifications (2) are also found by tatajuba. The correspondence between the changes found in (2) and in tatajuba are shown in Table 3. Some (2 out of 12) are missing since we limited the search to homopolymers of length 3 or higher. Interestingly, tatajuba misses the changes originally reported in locations 1268739 and 1268827 from 3A to 2A. Instead, it reports 3A for all samples, since it stores the distribution of homopolymers truncated at 3: even if the 2A (dimer) form is more frequent than the trimer 3A (and would therefore be the consensus), tatajuba does not keep track of dimers. Upon further inspection (by allowing dimers, results not shown) we confirmed this is the case for 1268739. For 1268827 and other cases where tatajuba does not detect the HT change for some samples, like locations 1268899 and 1268944, may be due to low tract coverage or missing context from raw reads (see also Figure 7 below for an example where there is insufficient context around a given HT).

**Table 3.** Correspondence between HT length modification found here and in (2)

Location in (2)	Location in tatajuba	change	Samples in Figure 4 where change is observed	Confirmed by tatajuba	Observations
846037 (pgp2)	846032	4 T > 3 T	*62–5*	Y	
846075 (pgp2)	846075	4 A > 3 A	*H2_P1_R1*	Y	
846256 (pgp2)	846255	4 A > 3 A	*50–1*	Y	*62–5* and *17–19* show variability
846319 (pgp2)	846317	3 A > 2 A	*H5_P4_R1*	Y	
846702 (pgp2)	846701	2 G > 4 G	*C_jejuni_M1cam_R88*	Y	
1268531 (pgp1)	1268530	8 A > 7 A	*C_jejuni_M1cam_R10, 81–176_R2*	Y	
1268531 (pgp1)	1268530	8 A > 9 A	*81116_R1*	Y	
1268739 (pgp1)	1268738	3 A > 2 A		N	All samples have length of 3 in tatajuba
1268827 (pgp1)	1268826	3 A > 2 A		N	All samples have length of 3 in tatajuba
1268899 (pgp1)	1268898	5 T > 4 T	*5–45*	Y/N	Not found in previously reported *17–48*; *5–45* is only partial
1268944 (pgp1)	1268943	4 A > 5 A	*H5_P2_R1*	Y/N	Tract is absent in some samples
1269149 (pgp1)	1269148	5 A > 4 A	*11168_R1*	Y	

The location shown corresponds to the beginning of the homopolymer, with tatajuba starting at zero (instead of one). Tract lengths smaller than 3 were not recorded and thus appear as ‘absent’.

For the *C. jejuni* data set, we found 5445 variants involving the HT sites from the VCF files generated by tatajuba. This number is well below the 41k variable tracts, since it does not account for missing tracts: HTs identical between the reference and the sample are not reported in the VCF file, as well as HTs which were not found in the sample. These variants generated a total of 10 115 functional annotations excluding upstream and downstream genes, describing 10 518 effects (Sequence Ontology terms). The most common annotations were intragenic gene (5031 variants), frameshift (2368), synonymous (1545), and missense (1018) variants. From those with high putative impact, besides frameshift variants we observed 107 variants with stop gained, 11 with start loss, and 6 with stop lost. It is also worth noting that a variable tract may be represented not by an indel but by a SNP in a sample, for instance ACA**TTTT**ACA and ACA**TTTG**ACA – which explains the synonymous variants detected above.

**Figure 5.**
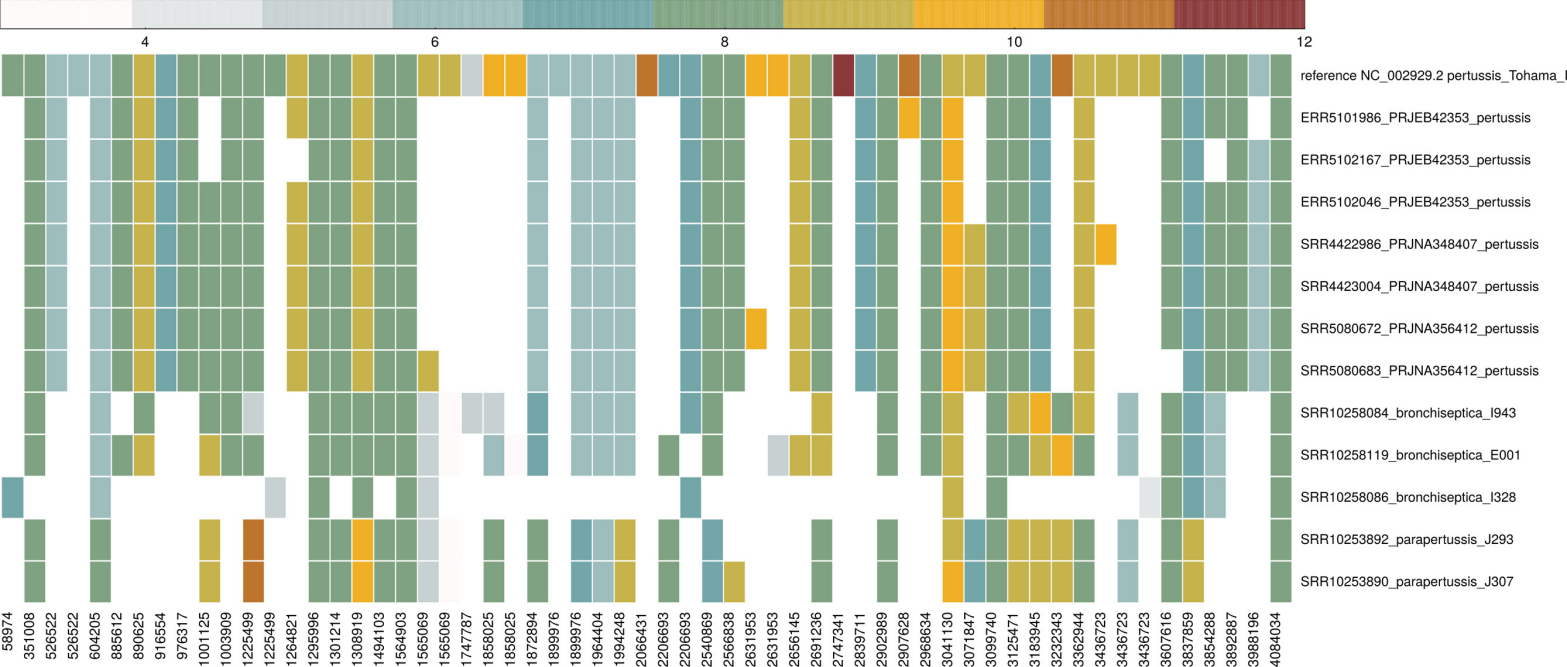
Average tract length for selected *Bordetella* samples, over regions reported in (15). The samples were arbitrarily selected for display purposes, to show the variety of tract lengths.

A previous study described 58 HTs in *B. pertussis* putatively involved in phase variation (15). This study employed a Markov model to find HTs longer than expected by chance, using three reference genomes: *B. pertussis*, *B. parapertussis* and *B. bronchiseptica*. We thus compared the previously identified genome locations with the closest equivalents as reported by tatajuba on our *Bordetella* dataset. The result is shown in Figure 5, where most originally reported HTs are found by tatajuba, 50 out of 58 when correcting for strand bias. From the originally reported HTs missing, with the exception of the HT at ORF BP0146, seven are found with our model once we relax the strand bias correction; this suggests that some HTs reported in the literature and in reference databases may be spurious. In (15), they consider the gene strand when annotating the start of the HT, but to make locations comparable, we report the HT’s location with respect to the leftmost base in the reference.

### Phylogenetic agreement

**Figure 6.**
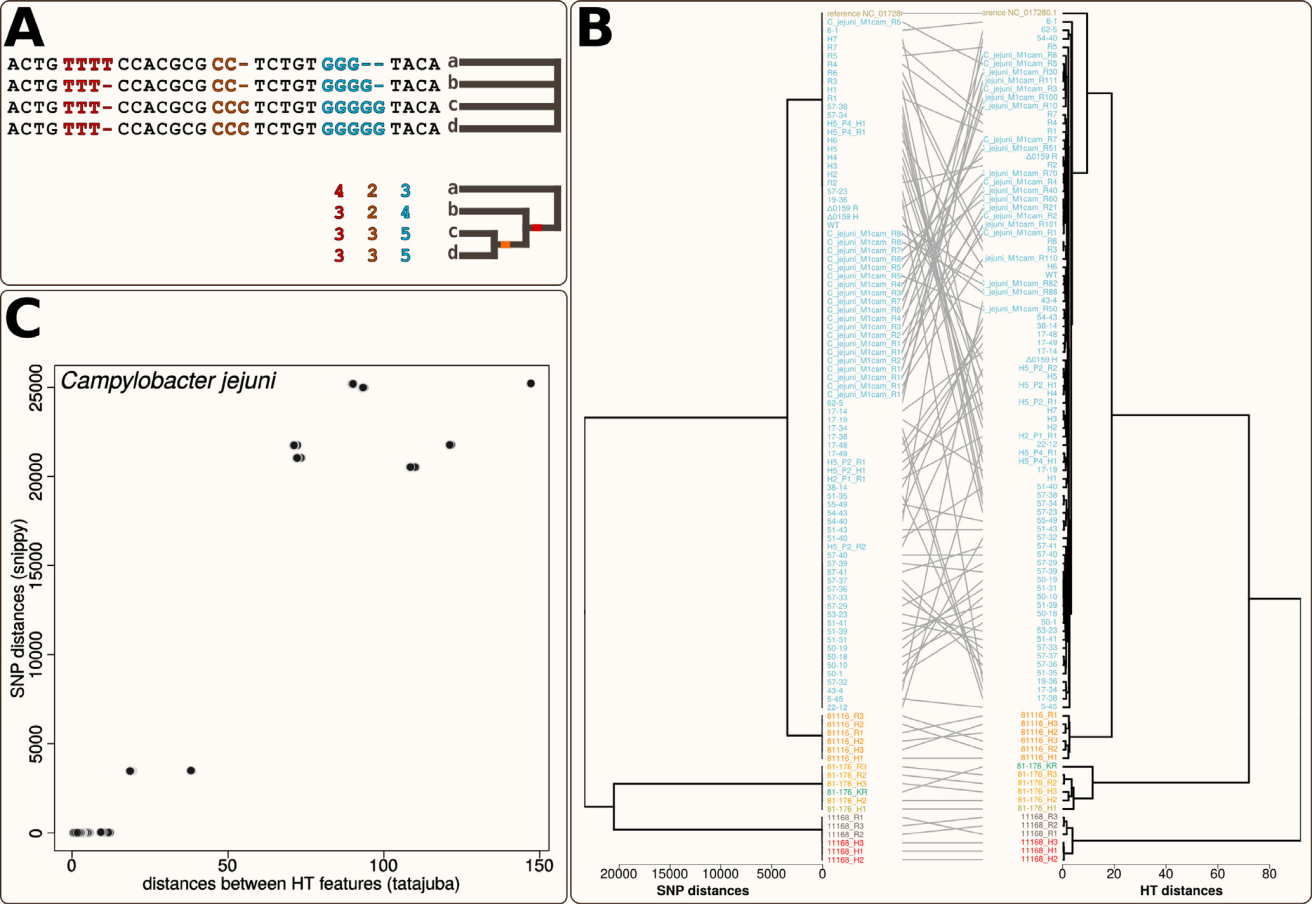
Comparison between sample distances using SNP differences or average HT length profiles for the *Campylobacter* data set. (**A**) Schematic example of four sequences a,b,c,d having zero SNPs (top) which nonetheless are different, once their homopolymeric tract lengths (coloured numbers) are taken into account. The coloured branches represent putative HT length changes. (**B**)Tanglegram comparing the UPGMA dendrograms from the distance matrices if we use the number of SNP differences (left) or the Euclidean distance between their average tract length profiles (right). The samples are coloured according to their HT-based clustering. (**C**) Direct comparison between the pairwise distance matrices, where each point represents an element of the matrix, i.e. a sample pair, using SNPs (y axis) or HTs (x axis).

Tatajuba is not a variant calling tool, and should not be used as a replacement for standard phylogenetic inference methods. However, there is an unexplored potential in the phylogenetic signal of homopolymeric tracts which can be explored with our tool. This is because a change in the HT length is represented as an indel in the alignment, and therefore is not properly modelled by most tree inference methods (11–13,27–29). In Figure 6A, we show a situation where the genomes do not have any polymorphic site (when considering only nucleotides), and thus have zero distance between each other. Since indels are treated as missing data by most phylogenetic methods, they cannot be used to distinguish between distinct topologies (11–13). However, if we account for the indel patterns within each HT (the coloured segments), then we can infer which genomes are more similar, assuming that HT-related indels are reliable, i.e. less likely to be alignment artefacts (10,30,31).

To show the effect of incorporating the HT signal on our data sets, we compared tatajuba with a traditional phylogenomic inference, using snippy to create the core genomes from both data sets. Snippy tracks all nucleotide variants with respect to a reference genome (same ones as used for tatajuba, in our case) while discarding insertions. While tatajuba identified 38 937 distinct HT start locations, snippy found 34 644 SNP locations, since it excludes insertions with respect to the reference. In Figure 6B, we see how tatajuba recovers the same clusters as snippy, but with higher resolution as represented by the longer branch lengths and fewer polytomies. This becomes evident when we compare directly the pairwise distances between samples using HTs and SNPs (Figure 6C): there is overall good correspondence, as expected, but especially for the most similar sample pairs, there is no observable SNP differences despite subtle differences in their HT lengths.

The same result was observed for the *Bordetella* sequences when we use one reference genome, including the paraphyly of *B. bronchiseptica* (results not shown). For this comparison there were 120 201 SNPs and 115 528 distinct variable HT locations. Notice that the number of distinct HT locations is smaller than the total number of variable HTs since some map to the same location (Table 2). The multi-genomic panel of references cannot be compared with snippy, however Figure 2 alone shows the phylogenetic improvement of using a panel instead of a single reference: the monophyly is recovered, and the longer branches further indicate a higher phylogenetic resolution (32).

## DISCUSSION

One future direction is to explore the HTs explicitly as phylogenetic markers (30), for instance by extending an alignment from their flanking regions, and combining it with a continuous trait model for the HT lengths (33). The HTs can be rapidly identified across samples, and as we observed carry evolutionary information, although we are only scratching the surface of its potential in this manuscript. By exhaustively exploring populations of genomes for their presence, we may find regions of phylogenetic importance. The software allows for multiple genome annotation and therefore can work with several reference genomes.

Tatajuba can be used to help infer the phenotypic effect of tract length variations, by finding those in coding regions and by describing the change in tract length — in coding regions, we expect frameshift mutations to have a higher impact than a tract length difference of multiples of three. Tatajuba outputs a BED file describing the location in the chromosomes involved in a homopolymeric tract, which can be used in standard workflows, with established variant callers. It also generates a VCF file for each sample, restricted to the HT regions and compatible with variant effect prediction tools.

Strand bias, where reads from the forward strand disagree with reads from the reverse strand, are more common around homopolymeric regions (34–36). Although less affected than other technologies, Illumina sequencing can generate spurious indels within HTs (37–39), especially for HT lengths longer than 14 bp (40,41), and it was estimated to affect up to 1% of the genes in a metagenomic data set (42). A recent survey showed that up to 5.3% of all Illumina errors are related to homopolymers of length 3 or more (43). To correct for the length errors induced by strand bias, tracts present in only one strand can be flagged (44), or a filter can be added to exclude length disparities associated with the strand (34). Tatajuba only considers tract lengths observed on both strands. It has been observed that error correction algorithms might introduce errors around HTs (5), although alternatives exist, in particular quality-score-based error removal (5,45).

BWA-aln is optimized for short reads and has very good performance when the read is similar to the reference genome except for HTs at the end of the alignment (46). However, it fails to map reads with lower matches, unlike mappers which can spuriously return random mappings (46). BWA-aln is thus well suited for tatajuba: in our case the HT is never at the end of the alignment, since it is flanked by conserved sequences, and we assume the presence of a close reference genome.

**Figure 7.**
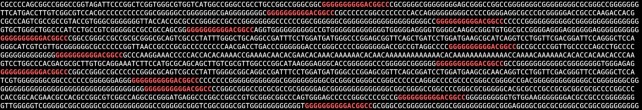
Search for a specific homopolymer tract using raw reads. The HT reported in (15) has 15 Gs in location 147886 of the *B. pertussis* Tohama I genome. A loose text search for sequence GGGGGGGGGGACGGCC (and its reverse complement GGCCGTCCCCCCCCCC) returns the reads above for five samples (ten paired end files), with the search text in red. Each sample read has fewer than five valid tracts.

It is important to keep in mind that our procedure is based on raw reads (samples), and reports HTs which were observed in these samples. This explains why tatajuba could not find the HT reported at BP0146 (genome location 174886) even without accounting for strand bias. In Figure 7, we show a list of all reads from five samples potentially containing the HT, as defined by a small stretch of the poly-G followed by a short flanking region of 6 bases. There we see that this HT is likely to be rejected by tatajuba since it does not have sufficient context (i.e. flanking regions) or its coverage depth is too low.

It is also important to note that tatajuba compares the HTs from each sample to a set of common reference genomes and thus the comparison between samples from different species is automatic, that is, we don’t need to map between reference genomes as in (15).

## CONCLUSION

HTs are widespread in many bacterial species, and variation in HT length can regulate gene expression. In both bacterial species examined here, HTs were found in large numbers, rendering the task of merely identifying such tracts unmanageable without automation. Clearly, with this number of HTs, an automatic/systematic way of investigating variation is required, which currently does not exist even for assembled genomes. Even when our analyses relied on a single reference genome, we show how we can have meaningful results when several species are analysed together. Tatajuba provides a huge scope for identifying potential genetic and therefore phenotypic variation which has thus far not yet been explored systematically. It therefore facilitates the discovery of important biological insights. Tatajuba cannot solve the coverage bias induced by some sequencing technologies towards HTs, but it excludes tracts with low depth and within reads without enough context, for instance those at the end of the read, without a flanking region.

Some sequencing platforms are sensitive to homopolymers, which can induce indel errors. For instance, MiSeq can find the correct HT length more often than the Ion Torrent PGM or the 454 GS Junior (37). When the HT leads to sequencing mistakes, reads from the forward strand may produce a HT length distinct from reads from the reverse strand – which would be summarized by tatajuba through the length distribution. We cannot fully eliminate systematic bias from sequencing and bioinformatics, but we can limit it. Additionally, tatajuba can be used as a quality control tool to identify these systematic bias issues. It should be used whenever any sequencing method, technology, or library preparation is being updated on a standard set of bacteria.

## DATA AVAILABILITY

Tatajuba is available under the open source GNU GPL 3 licence from https://github.com/quadram-institute-bioscience/tatajuba. The software is written in ANSI C (C11 standard with GNU extensions), validated using unit tests and packaged for autotools. The software is also available on bioconda, with Docker and singularity images. The samples used in this study are from public databases (e.g. https://www.ebi.ac.uk/ena), and are listed in the Supplementary Tables and in https://github.com/quadram-institute-bioscience/tatajuba/tree/master/docs.

## Supplementary Material

lqac003_Supplemental_Files
